# Effect of sequential delivery of 1‐ and 2‐MHz bipolar microneedling radiofrequency energy on thermal tissue reactions in a minipig model

**DOI:** 10.1111/srt.13898

**Published:** 2024-08-30

**Authors:** Sung Bin Cho, Sun Young Kang, Yea‐Jin Lee, Min Choi, Bora Kim, Jin‐Chul Ahn

**Affiliations:** ^1^ Yonsei Seran Dermatology and Laser Clinic Seoul South Korea; ^2^ R&D Center Shenb Co., Ltd Seoul South Korea; ^3^ Medical Laser Research Center College of Medicine Dankook University Cheonan South Korea

**Keywords:** alternating current, bipolar, frequency, gated pulse, parallel contact cooling, porcine model, radiofrequency, tissue reaction

## Abstract

**Background:**

Bipolar microneedling radiofrequency (RF) treatment generates different patterns of thermal reactions, depending on the skin impedance and RF treatment parameters, including the frequency, power, conduction time, settings of sub‐pulse packs, and penetrating depth and type of microneedles used. We compared the effect of sequential delivery of 1‐ and 2‐MHz bipolar RF energy to in vivo minipig skin on thermal tissue reaction.

**Methods:**

RF treatments at frequencies of 1 and 2 MHz were sequentially delivered to minipigs’ skin in vivo. A histological study was performed to analyze RF‐induced skin reactions at 1‐h and at 3‐, 7‐, and 14‐days post‐treatment.

**Results:**

The skin specimens demonstrated that the two different frequencies of RF treatment generated mixed patterns of the peri‐electrode coagulative necrosis (PECN) according to the experimental settings and tissue impedance. In the PECN zone, the tissue coagulation induced by the first RF treatment was surrounded by the effect of the later RF treatment at the other RF frequency. In the inter‐electrode non‐necrotic thermal reaction zone, the effect of the latter RF treatment was widespread and deep through the dermis, which had received RF treatment at the other frequency first. The delivery of pulsed‐type RF energy at sub‐pulse packs of 6 or 10 provided effective RF delivery over long conduction time without excessive thermal damage of the epidermis. Nonetheless, by sequential delivery of two different RF frequencies, RF‐induced tissue reactions were found to be markedly enhanced.

**Conclusion:**

The sequential delivery of 1‐ and 2‐MHz RF energy induces novel histological patterns of tissue reactions, which can synergistically enhance the thermostimulatory effects of each RF setting. Moreover, variations in patterns of tissue reactions can be generated by regulating the order of frequencies and the number of sub‐pulse packs of RF used.

## 1 INTRODUCTION

Bipolar, alternating current, microneedling radiofrequency (RF) treatment induces thermal reactions in the targeted areas of the skin. The degree of thermal reactions mainly depends on the RF‐induced tissue temperatures that ranges from non‐necrotic thermal reactions at temperatures of 40°C−45°C and coagulation necrosis at >60°C, to tissue desiccation at >100°C.^1^, ^2^ RF‐induced thermal reactions can be described according to the areas of tissue changes, including peri‐electrode and inter‐electrode areas.^3^, ^4^, ^5^, ^6^, ^7^, ^8^, ^9^, ^10^ Additionally, RF energy generates different patterns of thermal reactions depending on the skin impedance and RF treatment parameters, including the frequency, power, conduction time, and the penetrating depth and type of microneedles used.^4^, ^5^, ^6^, ^7^, ^8^, ^9^, ^10^, ^11^ Recently, the effects of sub‐pulse packs on RF‐induced tissue reactions have been reported.^3^, ^5^, ^12^ Various factors, including the number of sub‐pulse packs, on‐time of the sub‐pulse pack, off‐time between sub‐pulse packs, and total treatment time, which refers to the sum of total RF conduction time and total off‐time, can be regarded as significant variations during pulsed‐type RF treatment.^3^, ^12^

Generally, tissue reactions of coagulation necrosis and/or non‐necrotic thermal reactions can be observed in the peri‐electrode area after RF, whereas those of non‐necrotic thermal reactions can be found in the inter‐electrode area.^3^, ^4^, ^5^ Coagulation necrosis in the peri‐electrode area can be generated from the tips of microneedles when bipolar alternating current RF energy is delivered to areas of homogeneous tissue impedance.^6^ However, the location of generating a necrotic thermal reaction may differ along the peri‐electrode area in the layered tissues with abundant adnexal components presenting various levels of tissue impedance.^3^, ^4^ With the assumption that no desiccative tissue reactions occur in the peri‐electrode area, which would inhibit further propagation of RF pulses, non‐necrotic thermal tissue reactions are found in the inter‐electrode area, without marked tissue necrosis, as observed by microscopy.^3^

RF treatment at different frequency settings has been proven to result in similar, yet different patterns of thermal reactions.^10^, ^11^, ^12^ In this observational and descriptive study, we compared the effects of sequentially delivered 1‐ and 2‐MHz RF energy on RF‐induced early and late thermal reactions of the skin, using a bipolar, alternating current, microneedling RF device on minipig skin in vivo. Once the microneedles were inserted into the minipig's skin, RF treatment at two different frequencies (1 and 2 MHz) were sequentially delivered at 50‐ms intervals. Additionally, RF treatment at each frequency was delivered under various conditions, by regulating the number of sub‐pulse packs, total treatment time, RF conduction time, and total off‐time. Minipig skin specimens, which were obtained at 1‐h and 3‐, 7‐, and 14‐days after RF treatment, were then microscopically analyzed.

## 2 MATERIALS AND METHODS

### 2.1 In vivo minipig model

Four 10‐week‐old female minipigs (*Sus scrofa* domestica) were purchased (M‐pig^®^; CRONEX Inc., Cheongju, Korea), and the in vivo experiments were performed at the age of 12 weeks, when the pigs weighted 55−75 kg. Ethical approval for this study was obtained from the Ethics Committee of the Institutional Animal Care and Use Committee in CRONEX Inc. in Cheongju, Korea (CRONEX‐IACUC: 202210005). All animal experiments were performed in accordance with the guidelines of the National Institutes of Health Guidelines for the Care and Use of Laboratory Animals and the Institutional Animal Care and Use Committee.

General anesthesia was administered via an intramuscular bolus injection of tiletamine/zolazepam (5 mg/kg) and xylazine (2 mg/kg). Thereafter, we performed endotracheal intubation and connected the pig to a ventilator. The lungs were ventilated with oxygen, and anesthesia was maintained with 2% isoflurane. Intravenous hydration with normal saline was maintained through a superficial auricular vein (25 mL/h). After gentle removal of the hairs on the abdomen, the lesions were cleansed with a mild soap and 70% alcohol. The skin was marked using black ink to outline a grid for each experimental setting (15 mm × 15 mm/grid; 40 grids/minipig; total of 160 grids).

### 2.2 Bipolar RF device and experimental parameters

An invasive bipolar, alternating current, pulsed type, microneedling RF device (VIRTUE RF™; Shenb Co., Ltd., Seoul, Korea) was used at frequencies of 1 and 2 MHz to analyze RF tissue reactions in in vivo porcine skin. The device delivered RF energy through 36 insulated microneedle electrodes (24 K gold‐plated surgical stainless steel) in a 15‐mm × 15‐mm disposable tip (Deep RF™ 36 tip; Shenb Co., Ltd.). The electrodes within this tip were uniformly arranged in a 6 × 6 pattern, with the nearest distance between microneedles being 3 mm. Parallel contact cooling (PCC), using a cooled sapphire metal plate with thermoelectric elements, was adapted to protect the epidermis from thermal injury. This plate was integrated into the handpiece. The temperature of the chilled plate was regulated as 15°C± 0.5°C for this experiment.

**FIGURE 1:**
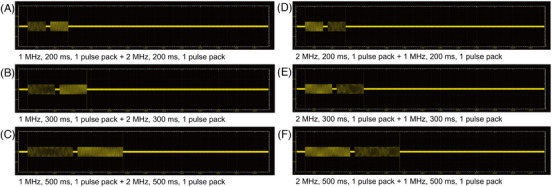
Schematic illustrations of experimental parameters for sequential invasive delivery of 1‐ and 2‐MHz bipolar radiofrequency (RF) energy on in vivo minipig skin tissue. Experimental settings of the frequency of 1 MHz, RF conduction time of (A) 200, (B) 300, and (C) 500 ms, and a single pulse pack (1‐200/300/500‐1) and additional RF treatment at 2 MHz and the single pulse pack of corresponding RF conduction time settings (2‐200/300/500‐1) with the interval settings between the two types of RF frequencies at 50 ms. Likewise, 2‐MHz RF treatment was followed by 1‐MHz RF treatment at the settings of (D) 2‐200‐1 + 1‐200‐1, (E) 2‐300‐1 + 1‐300‐1, and (F) 2‐300‐1 + 1‐300‐1.

In this study, RF treatment at frequencies of 1 and 2 MHz was performed on each grid of the experimental minipigs at a power of 35 W and an electrode penetration depth of 1.5 mm in one pass. Once the microneedles were inserted into the minipig's skin, RF treatment at the settings of the frequency of 1 MHz, with an RF conduction time of 200, 300, and 500 ms, and a single pulse pack (1‐200/300/500‐1), and additional RF treatment at 2 MHz, with an RF conduction time of 200, 300, and 500 ms, and a single pulse pack (2‐200/300/500‐1) were sequentially delivered, with the interval settings between the two types of RF frequencies being 50 ms (Figure 1A–F). Likewise, RF treatment at 2‐200/300/500‐1 and additional RF treatment at 1‐200/300/500‐1 were sequentially administered at 50‐ms intervals.

**FIGURE 2:**
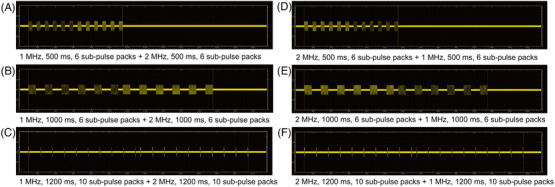
Schematic illustrations of experimental parameters for sequential delivery of 1‐ and 2‐MHz, pulsed‐type RF energy to in vivo minipig tissue. RF treatment at a frequency of 1‐MHz was followed by RF treatment at 2‐MHz, with sub‐pulse pack settings of 6 or 10. (A) 1‐500‐6 + 2‐500‐6, (B) 1‐1000‐6 + 2‐1000‐6, and (C) 1‐1200‐10 + 2‐1200‐10. Moreover, 2‐MHz RF treatment was followed by 1‐MHz RF treatment at settings of (D) 2‐500‐6 + 1‐500‐6, (E) 2‐1000‐6 + 1‐1000‐6, and (F) 2‐1200‐10 + 1‐1200‐10.

Secondly, RF treatment at 1 MHz, for a total treatment time of 500 ms (total RF conduction time, 250 ms and total off‐time, 250 ms) and 1000 ms (total RF conduction time, 500 ms and total off‐time, 500 ms) with six sub‐pulse packs (1‐500/1000‐6), and additional RF pulses at 2 MHz, for a total treatment time of 500 and 1000 ms with six sub‐pulse packs (2‐500/1000‐6) were sequentially delivered at 50‐ms intervals (Figure 2A–F). The total treatment time referred to the sum of the total RF conduction time and the total off‐time. Likewise, RF treatment at 2‐500/1000‐6 and additional RF pulses at 1‐500/1000‐6 were sequentially delivered at 50‐ms intervals. Additionally, RF treatment at 1‐1200‐10 (total RF conduction time, 120 ms, and total off‐time, 1080 ms) and additional RF pulses at 2‐1200‐10 were sequentially delivered at 50‐ms intervals. Similarly, RF pulses at 2‐1200‐10 and additional RF treatment at 1‐1200‐10 were sequentially delivered at 50‐ms intervals.

**FIGURE 3:**
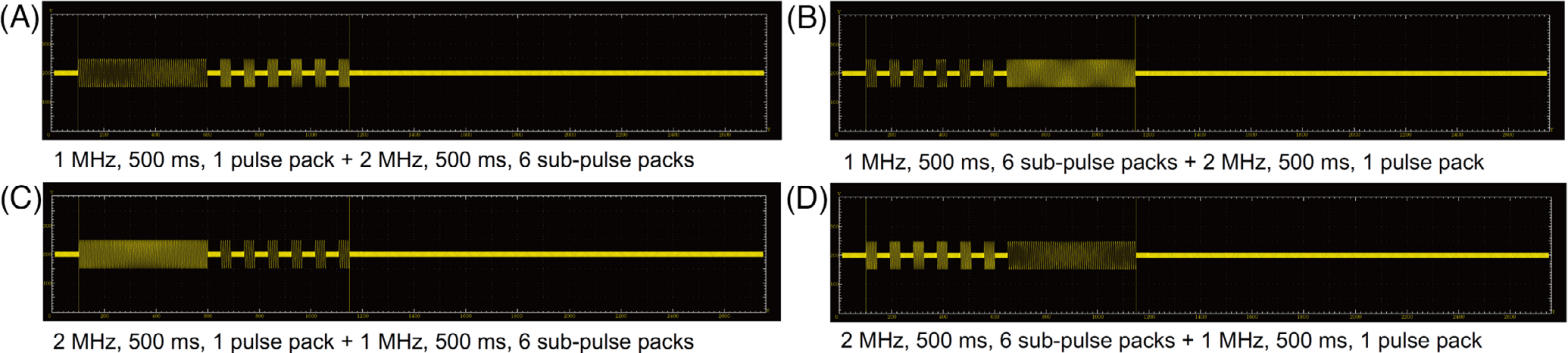
Schematic illustrations of experimental parameters for sequential delivery of 1‐ and 2‐MHz RF at various sub‐pulse pack settings to in vivo minipig tissue. RF treatment at a frequency of 1‐MHz was followed by that at 2 MHz, with sub‐pulse pack settings of 1 or 6 (A) 1‐500‐1 + 2‐500‐6 and (B) 1‐500‐6 + 2‐500‐1. Moreover, 2‐MHz RF treatment was followed by 1‐MHz RF treatment at settings of (C) 2‐500‐1 + 1‐500‐6 and (D) 2‐500‐6 + 1‐500‐1.

Thirdly, RF treatment at 1‐500‐1 and additional RF pulses at 2‐500‐6 were sequentially delivered at 50‐ms intervals (Figure 3A–D). RF treatment at 2‐500‐1 and additional RF treatment at 1‐500‐6 were sequentially delivered at 50‐ms intervals. Furthermore, RF treatment at 1‐500‐6 and additional RF treatment at 2‐500‐1 were sequentially delivered at 50‐ms intervals. RF treatment at 2‐500‐6 and additional RF treatment at 1‐500‐1 were also sequentially delivered at 50‐ms intervals. Neither pretreatment topical anesthesia nor pre‐ and post‐cooling was applied. No prophylactic topical or systemic antibiotics or steroids were used after RF treatment.

### 2.3 Histological evaluation

The experimental minipigs were euthanized to sample the treated tissue in a humane manner, according to standard protocols. One hour and 3, 7, and 14 days after treatment, full‐thickness tissue specimens, including the epidermis, dermis, and subcutaneous fat were obtained for microscopic evaluation. Each sample was fixed in 10% buffered formalin and was embedded in paraffin. Then, serial tissue sections of 4‐µm thickness for each treatment setting were prepared and were stained with hematoxylin and eosin.

## 3 RESULTS

### 3.1 Effect of sequential 1‐ and 2‐MHz, single pulse pack RF delivery

**FIGURE 4:**
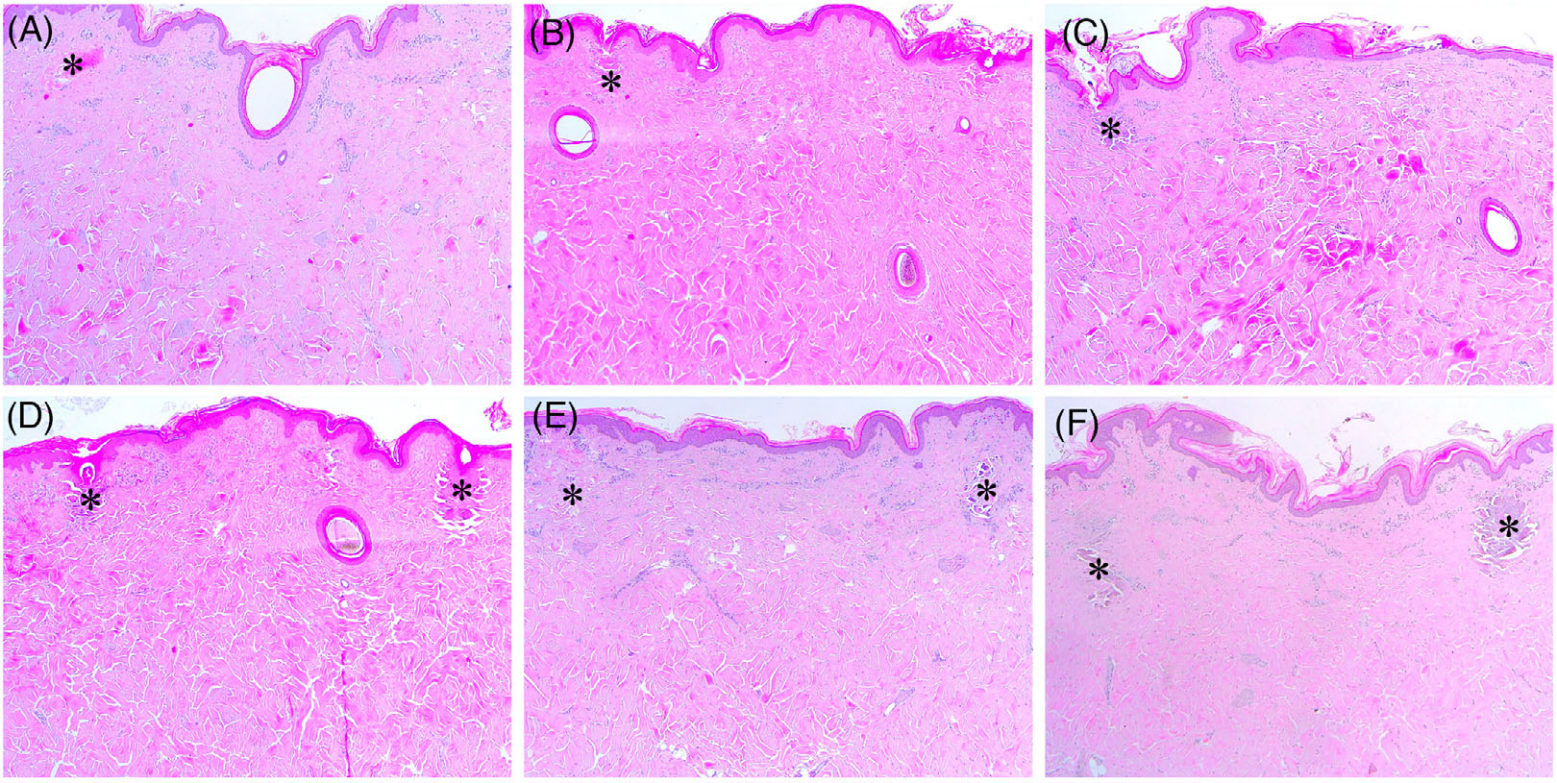
Immediate tissue reactions in in vivo minipig skin after sequential 1‐ and 2‐MHz RF delivery. The skin of a minipig was treated with RF at the settings of (A) 1‐200‐1 + 2‐200‐1, (B) 1‐300‐1 + 2‐300‐1, (C) 1‐500‐1 + 2‐500‐1, (D) 2‐200‐1 + 1‐200‐1, (E) 2‐300‐1 + 1‐300‐1, and (F) 2‐500‐1 + 1‐500‐1. Asterisks, peri‐electrode coagulative necrosis zones. Hematoxylin and eosin staining. Original magnification × 40.

One hour after RF treatment at 1‐200/300/500‐1 + 2‐200/300/500‐1, a well‐demarcated round to oval‐shaped peri‐electrode coagulation necrosis (PECN) zone was generated at the proximal part of the penetrating electrode in the upper papillary dermis of the minipig (Figure 4A–C). The PECN zone was histologically presented as a 1‐MHz RF‐induced, round to oval‐shaped relatively basophilic tissue coagulation, which was surrounded by a 2‐MHz RF‐induced notably eosinophilic tissue coagulation. The size of the PECN area became larger as the duration of RF conduction time increased. The inter‐electrode non‐necrotic thermal reaction (IENT) zone was markedly present in the upper papillary, mid to deep dermis, and in subcutaneous fat layers, without noticeable coagulation necrosis. Histologically, the IENT zone in the dermis was composed of 1‐MHz RF‐induced diffusely edematous thermal reactions and 2‐MHz RF‐induced multiple focal eosinophilic areas of high‐degree, but non‐coagulative, thermal reactions. The areas of 2‐MHz RF‐induced tissue changes were found in the dermis around the microneedles at a shorter conduction time, and around as well as between the microneedles at a longer conduction time.

One hour after RF treatment at 2‐200/300/500‐1 + 1‐200/300/500‐1, the well‐demarcated round to oval‐shaped PECN zone was found at the proximal part of the penetrating electrode in the upper papillary dermis (Figure 4D–F). The PECN zone was presented as a 2‐MHz RF‐induced eosinophilic tissue coagulation region, which was surrounded by a 1‐MHz RF‐induced, round to oval‐shaped basophilic tissue coagulation region. The size of the PECN area became larger with an increasing duration of RF conduction time. Moreover, RF treatment at 2‐200/300/500‐1 + 1‐200/300/500‐1 generally showed a larger PECN region than did RF treatment at the corresponding conduction time settings of 1‐200/300/500‐1 + 2‐200/300/500‐1.

One hour after RF treatment at 2‐200/300/500‐1 + 1‐200/300/500‐1, an intensely eosinophilic IENT was generally found around the penetrating electrodes. Other areas of IENT presented wide and homogeneous thermal tissue changes in the upper and mid to deep dermis. Therefore, 2‐200/300/500‐1 + 1‐200/300/500‐1 generated more remarkable PECN than did 1‐200/300/500‐1 + 2‐200/300/500‐1. Moreover, 1‐200/300/500‐1 + 2‐200/300/500‐1 treatment generated more intense and widely distributed IENT than did 2‐200/300/500‐1 + 1‐200/300/500‐1 treatment. Thus, 1‐MHz RF pretreatment could more effectively enhance the IENT of subsequent 2‐MHz RF treatment.

**FIGURE 5:**
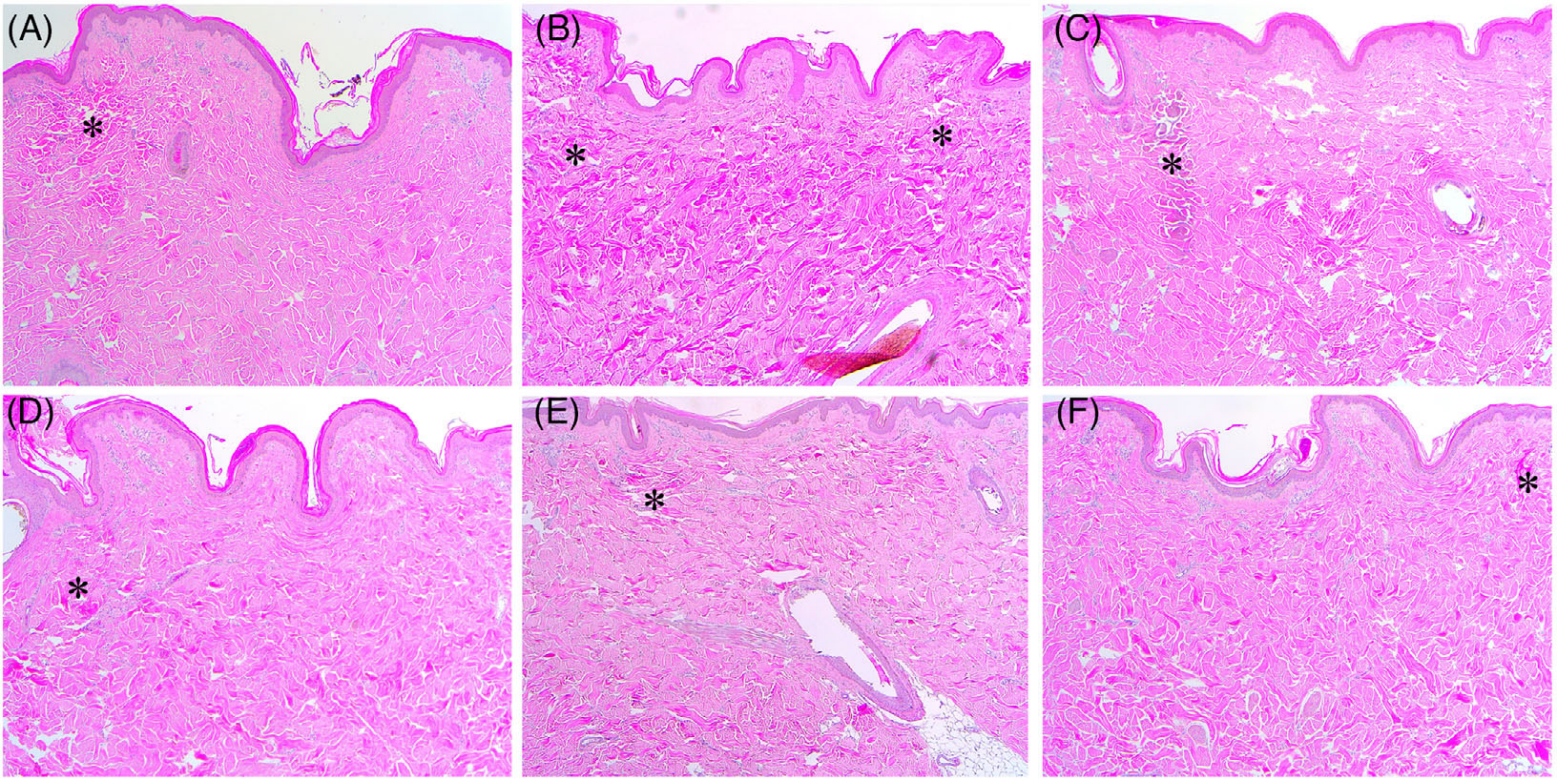
Tissue reactions in in vivo minipig skin after sequential 1‐ and 2‐MHz RF delivery on Day 7. The skin of minipig was treated with RF at the settings of (A) 1‐200‐1 + 2‐200‐1, (B) 1‐300‐1 + 2‐300‐1, (C) 1‐500‐1 + 2‐500‐1, (D) 2‐200‐1 + 1‐200‐1, (E) 2‐300‐1 + 1‐300‐1, and (F) 2‐500‐1 + 1‐500‐1. Asterisks, peri‐electrode coagulative necrosis zones. Hematoxylin and eosin staining. Original magnification × 40.

On days 7 and 14 after RF treatment, the histological features of PECN were still found in some serial section preparations, which were treated at 1‐200/300/500‐1 + 2‐200/300/500‐1 or 2‐200/300/500‐1 + 1‐200/300/500‐1 (Figure 5A–F). Additionally, notable histological features of collagen regeneration in the IENT zones were found under all experimental conditions. Nonetheless, the histological patterns of neocollagenesis were more homogeneous and widely distributed over the entire dermis under the experimental settings of 1‐200/300/500‐1 + 2‐200/300/500‐1 than under settings of 2‐200/300/500‐1 + 1‐200/300/500‐1. Furthermore, the experimental settings of 2‐200/300/500‐1 + 1‐200/300/500‐1 generated more intense, but non‐homogeneous patterns of neocollagenesis, which were distributed in the upper dermis and mid dermis, as compared to 1‐200/300/500‐1 + 2‐200/300/500‐1.

### 3.2 Effect of sequential 1‐ and 2‐MHz, multiple sub‐pulse pack RF delivery

**FIGURE 6:**
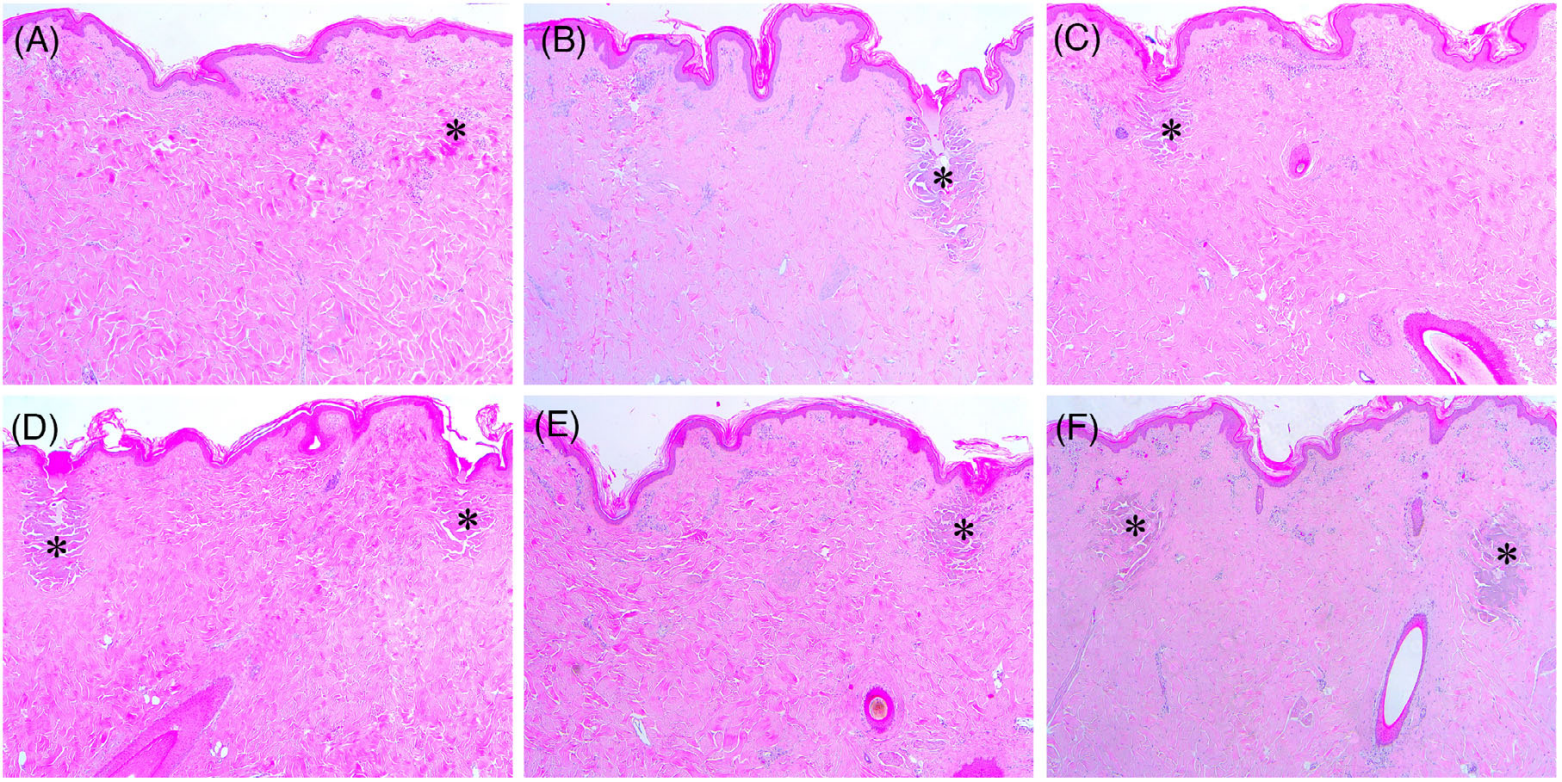
Immediate tissue reactions in in vivo minipig skin after sequential 1‐ and 2‐MHz, pulsed‐type RF delivery. The skin of a minipig was treated with RF at the settings of (A) 1‐500‐6 + 2‐500‐6, (B) 1‐1000‐6 + 2‐1000‐6, (C) 1‐1200‐10 + 2‐1200‐10, (D) 2‐500‐6 + 1‐500‐6, (E) 2‐1000‐6 + 1‐1000‐6, and (F) 2‐1200‐10 + 1‐1200‐10. Asterisks, peri‐electrode coagulative necrosis zones. Hematoxylin and eosin staining. Original magnification × 40.

The histological features of minipigs treated with RF at 1‐500‐6 + 2‐500‐6 included none to small PECN zones, with remarkable IENT zones, which were distributed superficial‐to‐deep in the dermis (Figure 6A). The mixed 1‐MHz followed by 2‐MHz RF‐induced eosinophilic tissue reactions were found in wide areas of the dermis between the electrodes. When RF was delivered at 1‐1000‐6 + 2‐1000‐6, a few serial sections demonstrated marked PECN in the upper and mid dermis, with IENT areas widely distributed throughout the dermis (Figure 6B). RF‐treatment at 1‐1200‐10 + 2‐1200‐10 exhibited a smaller area of PECN than did RF treatment at 1‐1000‐6 + 2‐1000‐6 (Figure 6C). Nonetheless, similar patterns of IENT were found among the RF settings of 1‐500‐6 + 2‐500‐6, 1‐1000‐6 + 2‐1000‐6, and 1‐1200‐10 + 2‐1200‐10.

**FIGURE 7:**
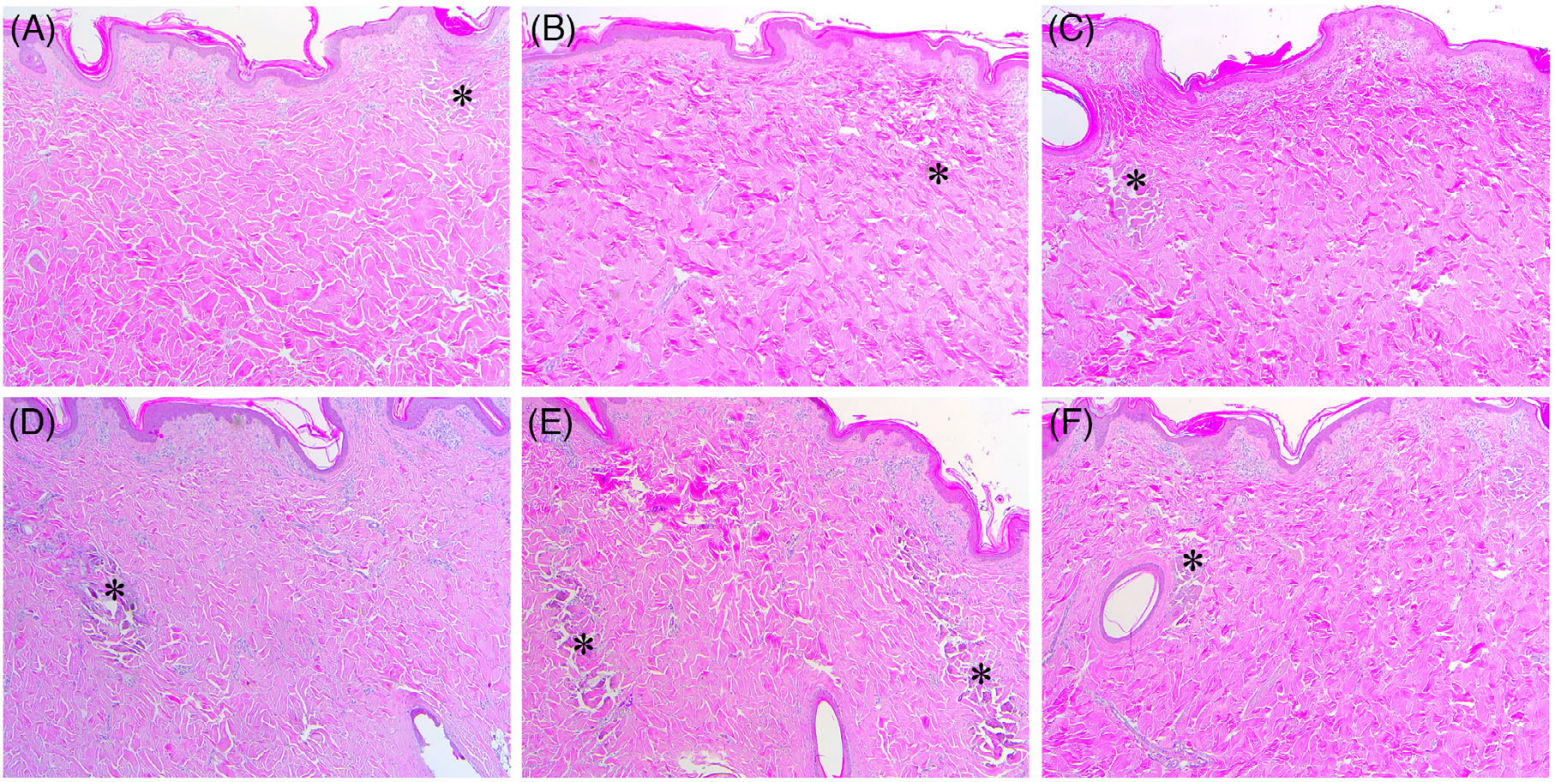
Tissue reactions in in vivo minipig skin by day 3 after sequential 1‐ and 2‐MHz, pulsed‐type RF treatment delivery on day 3. The skin of a minipig was treated with RF at the settings of (A) 1‐500‐6 + 2‐500‐6, (B) 1‐1000‐6 + 2‐1000‐6, (C) 1‐1200‐10 + 2‐1200‐10, (D) 2‐500‐6 + 1‐500‐6, (E) 2‐1000‐6 + 1‐1000‐6, and (F) 2‐1200‐10 + 1‐1200‐10. Asterisks, peri‐electrode coagulative necrosis zones. Hematoxylin and eosin staining. Original magnification × 40.

Moreover, RF treatment at 2‐500‐6 + 1‐500‐6 generated PECN areas in the upper dermis, which were notably larger and deeper at settings of 2‐1000‐6 + 1‐1000‐6 and 2‐1200‐10 + 1‐1200‐10, and with a higher degree of tissue coagulation (Figure 6D–F). Compared to the RF treatment settings of 2‐200/300/500‐1 + 1‐200/300/500‐1, sequential 2‐MHz RF delivery followed by 1‐MHz RF at the settings of multiple sub‐pulse packs generated widely distributed, marked IENT in the entire dermis. The early histological patterns of sequential 1‐ and 2‐MHz RF delivery at multiple sub‐pulse packs on Day 3 were similarly distinguishable among experimental settings compared with immediate histological patterns (Figure 7A–F). Nonetheless, histologically remarkable PECNs were more readily found in the experimental settings of 2‐MHz RF treatment followed by 1‐MHz RF treatment on Day 3.

**FIGURE 8:**
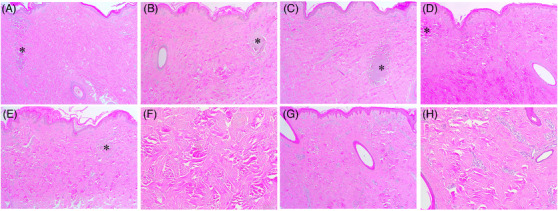
Tissue reactions in in vivo minipig skin at days 7 and 14 after sequential 1‐ and 2‐MHz, pulsed‐type RF delivery. The skin of a minipig was treated with RF at the settings of (A, B) 1‐1000‐6 + 2‐1000‐6, (C, D) 1‐1200‐10 + 2‐1200‐10, (E, F) 2‐1000‐6 + 1‐1000‐6, and (G, H) 2‐1200‐10 + 1‐1200‐10. (A, C) Reactions were noted at 7 and (B, D, E–H) 14 days after sequential 1‐ and 2‐MHz, pulsed‐type RF treatment. Asterisks, peri‐electrode coagulative necrosis zones. Hematoxylin and eosin staining. Original magnification (A–E, G) × 40, (F, H) × 100.

On days 7 and 14 after RF treatment, the histological features of PECN were more notably found in the specimens that were treated at 1‐1000‐6 + 2‐1000‐6 and 1‐1200‐10 + 2‐1200‐10, as compared to that in other treatment settings with multiple sub‐pulse packs (Figure 8A–D). Notable histological features of collagen regeneration in the IENT zones were found under all experimental conditions. On day 14, sequential 1‐MHz RF delivery followed by 2‐MHz RF generated diffuse areas of neocollagenesis in the entire dermis, and the degree of which seemed to be more remarkable depending on the overall RF treatment time. Moreover, sequential 2‐MHz RF delivery followed by 1‐MHz RF treatment resulted in 2‐MHz, multiple sub‐pulse pack‐induced polymorphous areas of neocollagenesis on day 14. These tissue reactions appeared to be enhanced by subsequent 1‐MHz RF delivery (Figure 8E–H).

### 3.3 Effect of sequential 1‐ and 2‐MHz, single pulse, and multiple sub‐pulse pack RF delivery

**FIGURE 9:**
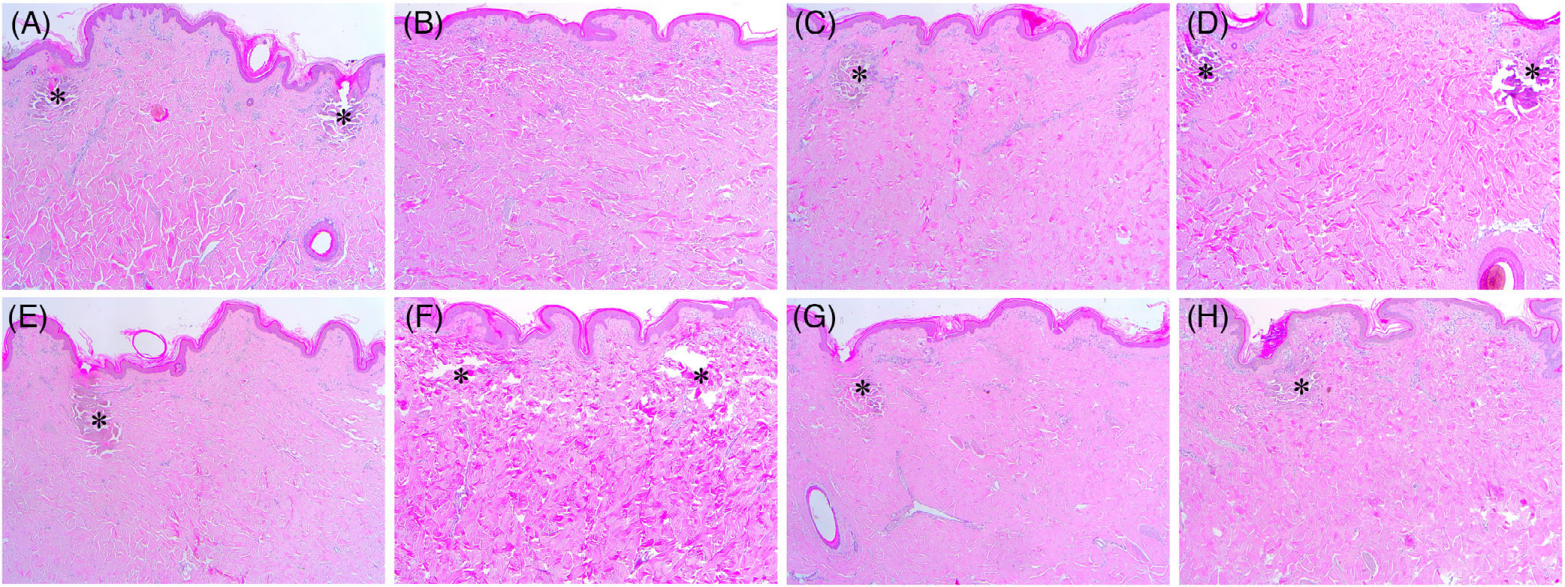
Immediate and early tissue reactions in in vivo minipig skin after sequential 1‐ and 2‐MHz RF delivery at various sub‐pulse pack settings. The skin of a minipig was treated with RF at the settings of (A, B) 1‐500‐1 + 2‐500‐6, (C, D) 1‐500‐6 + 2‐500‐1, (E, F) 2‐500‐1 + 1‐500‐6, and (G, H) 2‐500‐6 + 1‐500‐1. The skin specimens were obtained (A, C, E, G) immediately and (B, D, F, H) 3 days after RF treatment. Asterisks, peri‐electrode coagulative necrosis zones. Hematoxylin and eosin staining. Original magnification × 40.

The minipig skin specimen, which was obtained after RF treatment at a setting of 1‐500‐1 + 2‐500‐6, exhibited 1‐MHz RF‐induced, predominantly basophilic round to oval PECN lesions with 2‐MHz RF‐induced, fractionated eosinophilic coagulation of the collagen bundles. In these lesions, notable RF‐induced, diffusely edematous thermal reactions with eosinophilic tissue reactions were also found in the mid to deep dermis between the microneedles. Furthermore, RF treatment at a setting of 1‐500‐6 + 2‐500‐1 generated noticeable, but smaller PECN areas than did that at a setting of 1‐500‐1 + 2‐500‐6. Nonetheless, 2‐MHz RF‐induced, necrotic thermal reactions, which surrounded the 1‐MHz RF‐induced, basophilic PECN areas, produced high‐degree thermal tissue reactions that were more apparent in the specimens at 3‐days post‐RF (Figure 9A–D). Moreover, RF‐induced, multiple, small eosinophilic patches of high‐degree, but non‐coagulative, thermal reactions, were found in the upper, mid, and deep dermis around and between the microneedle insertions.

In the 2‐500‐1 + 1‐500‐6 experimental setting, narrow and cylindrical PECN zones were generated in the upper dermis, which were surrounded by basophilic necrotic collagen bundles (Figure 9E). In these regions, thermal reactions in the dermis seemed to be less marked than those obtained with other experimental settings. Nonetheless, RF‐induced non‐necrotic tissue reactions were more notable in 3‐day post‐RF specimens over all the layers of the dermis (Figure 9F). RF treatment at 2‐500‐6 + 1‐500‐1 also generated oval to cylindrical PECN zones, which were composed of central 2‐MHz RF‐induced, erythematous necrosis with surrounding 1‐MHz RF‐induced, basophilic coagulation areas (Figure 9G, H). Although the PECN areas under settings of 2‐500‐6 + 1‐500‐1 were smaller and less necrotic than those generated under 2‐500‐1 + 1‐500‐6 settings, the immediate IENT zones were more apparent in the specimens treated with RF at 2‐500‐6 + 1‐500‐1.

## 4 DISCUSSION

In this experimental pilot study, we histologically evaluated the effects of 1‐ and 2‐MHz RF energy sequentially delivered to the skin using a bipolar, alternating current, microneedling RF device, under various experimental settings differing in terms of the number of sub‐pulse packs, total treatment time, RF conduction time, and total off‐time for each frequency. The two different frequencies generated mixed patterns of PECN, varying with the experimental settings and tissue impedance. In the PECN zone, the tissue coagulation induced by the first RF treatment at one frequency was surrounded by the effect of the later RF treatment at the other RF frequency. In the IENT zone, the effect of the latter RF treatment was widespread and deep through the dermis that had undergone initial RF treatment at the other frequency. The delivery of pulsed‐type RF energy at sub‐pulse packs of 6 or 10 provided effective RF delivery over long conduction time, without causing excessive thermal damage in the epidermis. Nonetheless, sequential delivery of two different RF frequencies resulted in more marked RF‐induced tissue reactions.

In previous studies, RF treatment at a frequency of 0.5 or 1 MHz generated PECN zone with water drop‐ or chandelier‐like appearance in the upper or mid dermis, depending on the impedance of the target tissue.^10^, ^11^, ^15^ RF treatment at 2 MHz resulted in narrower, but more intensely injured PECN areas than did RF at other frequency settings.^10^, ^11^, ^15^ Moreover, RF treatment at a frequency of 0.5 or 1 MHz generated diffuse, edematous IENT areas in the dermis, whereas 2‐MHz RF treatment demonstrated notable thermal changes of the collagen bundles around the microneedles after a shorter conduction time, which propagated toward the paired microneedles with increasing conduction time.^10^, ^15^

In the present study, we found that sequential delivery of two different RF frequencies generated mixed patterns of PECN depending on the experimental settings and tissue impedance. The histological patterns of the PECN seemed to be composed of earlier RF‐induced tissue necrosis surrounded by later RF‐induced thermal reactions. When the former RF frequency was set to 1 MHz, round to oval, relatively basophilic PECN zones were found in the dermis, with a size larger than the PECN zones induced by 2 MHz RF. When the initial RF treatment was set to 2 MHz, oval to cylindrical, relatively eosinophilic PECN zones were formed in the dermis, and the degree of tissue degeneration seemed to be greater than that observed after 1 MHz treatment. Moreover, the histological features suggested that some PECN lesions in the upper to mid dermis, which corresponded to the level of the insulated part of the microneedles, could have resulted from accumulated thermal reactions induced by RF flow in the upper dermis with its high tissue impedance.

A gated delivery of RF energy reportedly generates wider ranges of non‐necrotic dermal tissue reactions with a lesser degree of coagulative thermal reactions and is achieved by increasing the number of sub‐pulse packs.^3^, ^5^ In this study, although the settings of total treatment time (500 ms) and six sub‐pulse packs delivered only 250 ms of RF energy. The experimental conditions of 1‐500‐1 + 2‐500‐6 notably presented 2‐MHz RF‐induced IENT throughout the dermal layers. We suggested that single‐pulse, 1‐MHz RF delivery initially generated the PECN and the widely distributed IENT in the dermis. Then, subsequent 2‐MHz RF treatment at multiple sub‐pulse‐settings generated lesser PECN and more marked IENT, along with the changes already present in the 1‐MHz pretreated dermal tissues. Furthermore, RF treatment at 2‐500‐1 + 1‐500‐6 generated cylindrically coagulated PECN with diffuse and homogenous IENT areas in the dermis. Interestingly, the experimental settings of total treatment time of 1200 ms and the number of sub‐pulse packs of 10, which delivered 120‐ms RF energy over 1200 ms, some specimen sections showed large areas of PECN with extensive thermal tissue reaction than did other experimental settings. We propose that those histological changes could have resulted from the cumulative RF‐induced thermal effects due to upward heat diffusion with enhanced impedance changes over a long treatment period.

In this study, we used PCC during RF treatment for all experimental settings to prevent excessive thermal injury to the epidermis and upper papillary dermis.^13^, ^14^ Skin cooling during energy‐delivering treatments has been adapted to reduce patients’ pain and post‐treatment adverse events.^13^, ^14^ Among the methods of skin cooling, including contact/non‐contact cooling and pre‐cooling/parallel cooling/post‐cooling, we opted to use PCC during RF treatments.^12^ Our research group has previously demonstrated the effects of PCC on RF‐induced thermal reactions of in vivo minipig skin by using thermometric and histological analyses.^15^ According to our previous study, PCC effectively protects the epidermis and upper papillary dermis against excessive thermal tissue injury, as compared to RF treatment without PCC.^15^ Furthermore, PCC markedly enhanced RF‐induced skin reactions in the mid to deep dermis at both early and late stages of wound healing and tissue regeneration.^15^ Because total treatment time settings for the sequential delivery of both frequencies in this study were relatively longer than those involved in previous studies, we decided to use PCC during these experiments to prevent thermal damage to the epidermis.^3^, ^4^, ^5^, ^6^, ^11^

The number of experimental RF parameters adjusted when delivering the treatment at two different frequencies were limited in this pilot study and the effects of RF treatment on the skin were only histologically evaluated in this study. Moreover, the amount of collagen fibers and appendages between minipig and human skin differ, which limits the extrapolation of our data to clinical situations for treating various dermatological conditions. Although our data exhibited no desiccative tissue reactions in the epidermis and dermis of the RF‐treated minipig skin specimens, high power and long RF treatment time settings were selected in our experiment to obtain histologically distinguishable differentiating points among RF settings. Therefore, when sequentially delivering two different RF frequencies to human skin, treatment parameters will need to be adjusted, depending on the skin reactions, to ensure safety and efficacy. Additionally, our data could not demonstrate representative thermal tissue reactions to treatment with all bipolar, alternating current, pulsed‐type RF devices. We consider that different patterns of RF‐induced tissue reactions could be obtained according to the type of device used, which have subtle technological differences.

## 5 CONCLUSION

In this study, we evaluated the effect of sequential delivery of 1‐ and 2‐MHz RF energy on early and late thermal tissue reactions in in vivo minipig skin using a bipolar microneedling RF device. Our data revealed that sequential delivery of RF treatment to the skin at two frequencies generated mixed patterns of the PECN according to the experimental settings and tissue impedance. In the PECN zone, the tissue coagulation induced by the initial RF treatment at one frequency was found to be surrounded by the thermal effect of the subsequent RF treatment at the other RF frequency. In the IENT zone, the effects of the second RF treatment were widely and deeply spread through the dermis after the first RF treatment at the other frequency. However, additional research using mixed RF treatment settings involving different frequencies and sub‐pulse packs are needed to confirm our findings. Moreover, controlled clinical studies are required before using sequential 1‐ and 2‐MHz RF delivery to human skin in clinical applications.

## CONFLICT OF INTEREST STATEMENT

The authors have no conflicts of interest or competing financial interests to declare.

## ETHICS STATEMENT

All experimental protocols were approved by the Ethics Committee of the Institutional Animal Care and Use Committee of CRONEX Inc., in Cheongju, Korea (CRONEX‐IACUC: 202210005).

## Data Availability

The data that support the findings of this study are available on request from the corresponding author.
